# A linear delay algorithm for enumerating all connected induced subgraphs

**DOI:** 10.1186/s12859-019-2837-y

**Published:** 2019-06-20

**Authors:** Mohammed Alokshiya, Saeed Salem, Fidaa Abed

**Affiliations:** 1 North Dakota State University, Fargo, ND 58102, USA; 2 University of Jeddah, Jeddah 23218, Saudi Arabia

**Keywords:** Biological networks, Subgraph enumeration, Reverse search

## Abstract

**Background:**

Real biological and social data is increasingly being represented as graphs. Pattern-mining-based graph learning and analysis techniques report meaningful biological subnetworks that elucidate important interactions among entities. At the backbone of these algorithms is the enumeration of pattern space.

**Results:**

We propose an efficient algorithm for enumerating all connected induced subgraphs of an undirected graph. Building on this enumeration approach, we propose an algorithm for mining all maximal cohesive subgraphs that integrates vertices’ attributes with subgraph enumeration. To efficiently mine all maximal cohesive subgraphs, we propose two pruning techniques that remove futile search nodes in the enumeration tree.

**Conclusions:**

Experiments on synthetic and real graphs show the effectiveness of the proposed algorithm and the pruning techniques. On enumerating all connected induced subgraphs, our algorithm is several times faster than existing approaches. On dense graphs, the proposed approach is at least an order of magnitude faster than the best existing algorithm. Experiments on protein-protein interaction network with cancer gene dysregulation profile show that the reported cohesive subnetworks are biologically interesting.

## Background

Mining interesting subgraphs from a large graph has been extensively studied. The modular structure has been observed in many real-world networks and shown to reveal insights into the intricate interactions that take place in real-world networks. Subgraph mining aims at discovering subgraphs that have interesting structural properties. Graph density, the ratio of present edges to the possible edges, has been the main property of interesting subgraphs. Abello et al. 2002 [1] proposed a greedy randomized algorithm for mining dense subgraphs. Matsuda et al. 1999 [2] introduced an approximation algorithm for mining a subset of the quasi-cliques present in a graph. A reverse-search-based algorithm for enumerating all dense subgraphs from an unweighted graph has been proposed in [3, 4].

Integrating node and edge attribute data with graph analysis has received attention since mining data from multiple sources has been shown to improve graph learning. In protein-protein interaction analysis, highly interacting proteins are more likely to form function modules. Functional module discovery can be aided by the integration of gene expression from multiple experiments as the genes in functional modules tend to have similar expression patterns [5, 6]. Moreover, subnetworks with differentially expressed genes have been shown to be good subnetwork biomarkers [6, 7]. Moser et al. [8] proposed the CoPaM algorithm for integrating the vertices’ attributes with dense subgraph mining. A reverse-search algorithm was used for mining dense cohesive subgraphs from a weighted protein-protein interaction network with nodes’ attributes have been proposed in [9]. Mining maximal homogeneous clique sets has been introduced in [10]. In Silva et al. [11], structural correlation mining was proposed for mining quasi-cliques that have correlated attributes.

In sparse attributed graphs, meaningful subgraphs can have very low density, yet exhibit high attribute similarity, e.g., biological pathways. Thus, it is important to mine connected subgraphs with high attribute similarity without the density constraint.

To achieve this goal, an algorithm for enumerating all connected induced subgraphs is needed as the backbone of the mining process. Additional attribute similarity constraints can be enforced while exploring the connected subgraphs search space. Moreover, the problem of enumerating all subgraphs is important in the field of computer-aided structure elucidation in cheminformatics for enumerating possible chemical graphs and stereoisomers [12]. The problem of enumerating all connected subgraphs might seem intractable since the number of these subgraphs can be exponential. However, in sparse graphs, the number of connected vertex sets is much smaller than the size of the power set of the set of vertices.

### Related work

The naive brute force algorithm to solve this problem is to generate the power set of the vertices, and then remove the elements in the power set that does not represent a connected subgraph. Clearly this algorithm is inefficient since it generates the power set of vertices, most of which are not connected. Another brute fore algorithm to solve the problem is to generate only connected subgraphs. The algorithm starts with one vertex as a subgraph, and then adds a neighbouring vertex to it every time. The process ends if the extended sub-graph is already visited or there is no more vertices to add. The drawback of this approach is storing all visited subgraphs which can be exponential and searching for the presence of a subgraph each time we extend the subgraph. Maxwell et al. [13] introduced the BDDE algorithm for enumerating all connected induced subgraphs. The BDDE algorithm follows a breadth-first discovery, and depth-first extension to enumerate the subgraphs. The algorithms starts with one vertex every time, and enumerate the binomial tree of neighbours of that vertex. This will enumerate all subgraphs that consist of the chosen vertex and its neighbors. The next step will be to enumerate subgraphs beyond the direct neighbors of the chosen vertex, by following each path in the binomial tree and treating it as a local search node, and building a sub-binomial tree for the direct neighbors of all vertices in the path except the vertices that are already visited. All neighbors of a local search tree are marked as visited before recursively call the depth-first search function, that eliminates duplicates that might be generated if the same neighbors are reached again by continued depth search. For a complete graph, the BDDE algorithm could consume a total space of *O*(2^*N*−1^), where *N* is the number of vertices in the input graph. Constraints defined over the nodes’ attributes can be integrating into the BDDE algorithm. Recently, the TGE algorithm for enumerating all induced connected subgraphs has been proposed [14]. This algorithm uses recursion to solve the problem. For a given vertex *v*, the connected vertex induced subgraphs are partitioned into two groups: subgraphs that include *v*, and subgraphs that do not include *v*. The connected vertex induced subgraphs included in the latter group can be enumerated by recursively solving the problem after deleting *v*. The former one can be solved again by partitioning the connected vertex induced subgraphs into two groups based on another chosen vertex. The author showed that the time complexity is *O*(1) for each solution by amortization.

Reverse Search is a powerful paradigm for enumeration. It was first introduced by Avis and Fukuda [15], and employed to solve several enumeration problems, including all induced connected subgraphs, spanning trees of a graph, maximal independent sets of a graph, and mining frequent bipartite episode from event sequences. The basic idea of Reverse Search is to arrange all subsets to be enumerated in a tree, where each node in the tree appears only once. The backbone of a reverse search algorithm is the definition of a *parent* operation that reduces a node to a unique parent node. By repeatedly applying the *parent* operation on any two different nodes in the search tree, they will be reduced to a shared canonical node, the root of the traversal tree. Once the *child* operation is defined by inverting the *parent* operation, we construct the enumeration tree by simply applying depth-first traversal, starting from the root.

A reverse search algorithm, RS-MST, for enumerating all induced connected subgraphs has been introduced in [15]. The parent operation employed for enumerating all induced connected subgraphs was based on the minimum spanning tree of the subgraph. For an induced connected subgraph, *G*, removing a vertex *v* that has a degree one in the minimum spanning tree of *G* cannot disconnect the subgraph. The authors in [15] proposed the child operation that reverses the vertex removal.

In this paper, we propose a novel reverse search algorithm for enumerating all induced connected subgraphs of a graph. Building on this enumeration approach, we propose an algorithm for mining all maximal cohesive subgraphs that integrates vertices’ attributes with subgraph enumeration. To efficiently mine all maximal cohesive subgraphs, we propose two pruning techniques that eliminate futile search subtrees in the enumeration tree, resulting in significant improvement in the running time of the algorithm. To demonstrate the effectiveness of the proposed algorithms and the pruning techniques, we conducted experiments on synthetic and real-world graphs.

## Methods

Let *G*=(*V*,*E*) be an undirected graph, where *V*={*v*_1_,...,*v*_*n*_} is the set of vertices, and *E*⊆*V*×*V* is the set of edges. For any vertex set *U*⊆*V*, let *G*(*U*)=(*U*,*E*(*U*)) denote the subgraph of *G* induced by *U*, whose edges include all the edges of *G* with endpoints in *U*. We call *U* a connected vertex set if *G*(*U*) is connected.

**Problem Definition:** Given an undirected graph *G*(*V*,*E*), enumerate all connected vertex sets, *CIS*(*G*).

$$CIS(G) = \{U \:|\: U \subseteq V \:and\: G(U)\: \text{is connected}\} $$

In this paper, we propose a linear-delay linear-space algorithm for enumerating all connected vertex sets of an undirected graph.

### Reverse search

In reverse search, a pattern extension rule defines how to generate child search nodes from a parent search node in the search space. The basic idea of reverse search is to arrange all solutions to be enumerated in a tree, rooted at an empty set node (canonical object), where each node in the tree appears only once under a specific parent node. In reverse search, a *parent* operation determines the unique parent node of a search node. This operation can be repeatedly applied on any two different nodes in the search tree until they reach a shared canonical node, the root of the traversal tree. Once the *parent* operation is defined, a *child* operation can be derived. Building on the parent-child operation, we build a tree-shaped traversal route on the set connected vertex sets. We perform the depth-first search on the tree without having the tree in memory to enumerate all induced connected subgraphs.

In this section, we define the *parent* operation and a data structure that allows for efficient *parent*/*child* operations.

### Parent child relationship

The following lemma is essential:

#### **Lemma 1**

If *G*(*U*) is a connected graph, *s*,*u*∈*U* are two distinct vertices, and *u* is the vertex with the largest shortest path from *s*, then *G*(*U*−*u*) is connected.

#### *Proof*

Assume that *u* is the furthest vertex away from *s* and deleting *u* results in a disconnected graph. This means that there exists at least one vertex *u*^′^ such that all paths between *s* and *u*^′^ go through *u*. So, the shortest distance between *s* and *u*^′^ is greater than the shortest distance between *s* and *u*. This contradicts our assumption that *u* is the vertex with the longest shortest path from *s* in *G*. Thus, *G*(*U*−*u*) is connected. □

Clearly, we can choose any vertex in *U*, then find the furthest vertex away from it and delete it, and still get a connected subgraph with size |*U*|−1. It does not matter which vertex to choose, and also does not matter if the chosen vertex has many vertices with the same furthest distance because deleting any of them will produce a connected subgraph. In this work, for defining a child/parent operation, we need to designate a vertex of the subgraph as the anchor vertex. We denote the vertex with the smallest vertex identifier (smallest vertex lexicographically) in *U* as *anchor*(*U*). Let *v*∈*U* be the vertex with the longest shortest path to *s*=*anchor*(*U*). If there are more than one vertex with the longest shortest path, we take the one with the largest vertex identifier. We refer to the vertex with the longest shortest path to *s* in a graph (*G*(*U*)) as the *utmost* vertex.

**Fig. 1:**
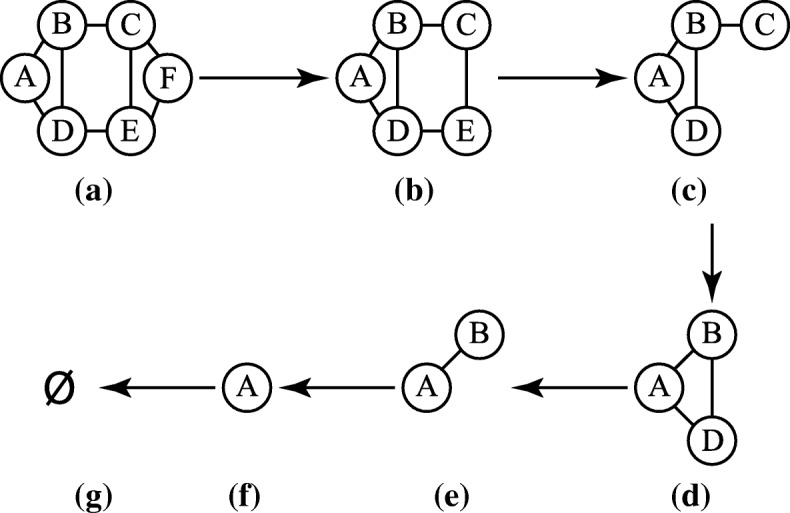
Applying the *parent* operation. Repeatedly applying the *parent* operation on a graph. **a** The *anchor* vertex is A, and the *utomst* vertex is F. **b** After deleting vertex F, vertices C and E become the furthest vertices with the same distance away from A, so E is the *utmost* vertex. We reduce the subgraph by deleting vertex E. **c**-**g** We apply the same procedure until deleting the last vertex A

We define the parent graph for a subgraph as follows: Let *G*(*U*) be a connected induced subgraph, *s*=*anchor*(*U*), and *v*∈*U* is the *utmost* vertex, then *G*(*U*−*v*) is the parent subgraph of *G*(*U*) (Lemma 1). The *parent* operation simply deletes the *utmost* vertex of a subgraph. It also can be repeatedly applied on a subgraph until reaching the canonical object (empty set). Figure 1 shows how to repeatedly apply the *parent* operation on a graph until reaching the empty set.

Now we derive the the *child* operation from the *parent* operation, as follow: Let *U* be a connected vertex set, *s*=*anchor*(*U*), *u*∈*U* is the *utmost* vertex of *U*, and *v*∈*V*∖*U* is connected to *U*. Then the subgraph induced by *U*^∗^=*U*∪{*v*} is a child of *G*(*U*) if and only if *v*>*s* (lexicographically) and one of the following conditions holds:

1. The distance from *s* to *v* is greater than the distance from *s* to *u*, or
2. Both *v* and *u* have the same distance to *s*, but *v* is lexicographically greater than *u*.

if *G*(*U*^∗^) is a valid child of *G*(*U*), we call *v* a valid candidate of *G*(*U*), otherwise, we call it an invalid candidate of *G*(*U*).

**Fig. 2:**
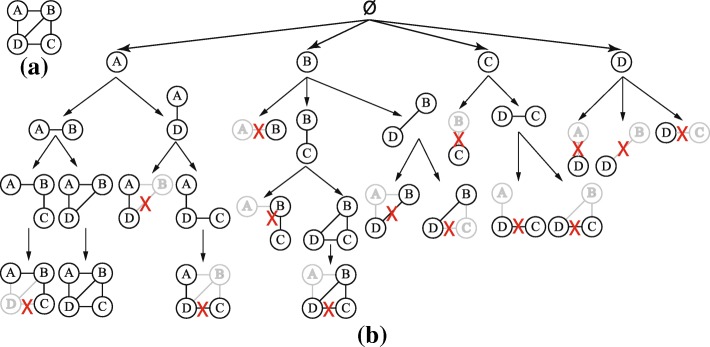
Sample graph enumeration. **a** Sample graph **b** Enumeration tree of the sample graph; the crossed search nodes indicate invalid subgraphs

Figure 2a shows a sample graph, and Fig. 2b shows the enumeration tree of this graph. Every search node in the enumeration tree represents a connected induced subgraph. Figure 2b shows that search node {*A*,*D*} is extended with vertex *C* to produce {*A*,*D*,*C*}; the other possibility {*A*,*D*,*B*} is crossed to indicate that it is not a valid child. In the leftmost branch, vertex *D* cannot be added to search node {*A*,*B*,*C*} because *distance*(*A*,*D*)=1<*distance*(*A*,*C*)=2. Under the subtree rooted at *B*, vertex *C* cannot be added to {*B*,*D*} because *distance*(*B*,*D*)=*distance*(*B*,*C*)=1, but *C* is lexicographically less than *D*. In the middle, search node {*B*,*A*} is crossed out because vertex *A* is less than the *anchor* vertex *B*.

### Distance-Array representation

One way to speed up Reverse Search is to design a data structure that speeds up testing for valid children. In this section, we describe a data structure to represent each subgraph to be enumerated, such that checking each valid child takes a constant time. Moreover, building the data structure of a valid child, given the data structure of the parent node, takes only *O*(*Δ*) where *Δ* is the maximum degree of the input graph.

Given a subgraph *G*=(*V*,*E*), we use a data structure of four arrays of size |*V*|. The *U* array holds the vertices of the subgraph in the same order they were visited. The *C* array holds the neighbors (candidates) of the subgraph. The *D* array holds the distance between the *anchor* vertex and all other vertices. And the *P* array keeps track of the parent of each vertex in *U* or in *C*; The parent of a vertex *v* is the vertex connected to it on the path to the *anchor* when *v* was first added to *C*. The *anchor* vertex does not have a parent vertex.

**Fig. 3:**
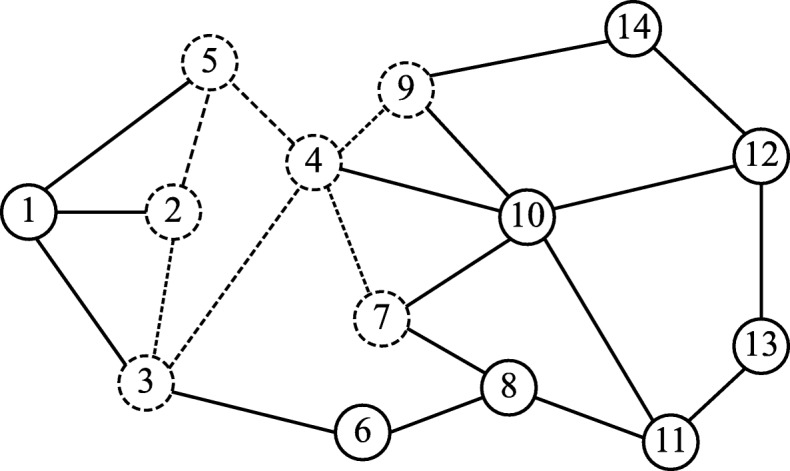
Extending a subgraph. A sample graph of 14 vertices and 22 edges. The dashed edges and vertices show the subgraph induced by the vertex set *U*={2,3,4,5,7,9}

Figure 3 shows a sample graph *G* of 14 vertices and 22 edges. The dashed vertices and edges represent the subgraph induced by the subset *U*={2,3,4,5,7,9}. The data structure for *U* is depicted in Table 1.

**Table 1:** Data Structure for *U*

U	2	3	5	4	7	9								
C	1	3	5	4	6	7	9	10	8	14				
v	1	2	3	4	5	6	7	8	9	10	11	12	13	14
D[v]	1	0	1	2	1	2	3	4	3	3	-	-	-	4
P[v]	2	-1	2	3	2	3	4	7	4	4	-	-	-	9

The *anchor* vertex of the induced subgraph *G*(*U*) is 2, and the *utmost* vertex is 9, with distance 3 away from the *anchor* vertex. The whole graph has 14 vertices labeled from 1 to 14. Only 6 vertices belong to the subset *U* and there are only five neighbors in *C*. Using this representation, we can easily determine the *anchor* vertex, since it is the first one in *U*, and the *utmost* vertex, since it is the last vertex in *U*. We can also get the distance between any neighbor of the subgraph and the *anchor* vertex in *O*(1) by accessing the corresponding index in *D*.

Using this representation, we can, for instance, extend the subgraph *G*(*U*) with the valid neighbor vertex *v*=10 to form the subgraph induced by the subset *U*^∗^={2,3,4,5,7,9,10}. We need to add neighbors of *v*=10 to *C* array and update their distances in *D* to be 4, and their parent in *P* to be 10. This will take only *O*(*Δ*). The data structure representation of *U*^∗^ is shown in Table 2.

**Table 2:** Data Structure for *U*∗

U	2	3	5	4	7	9	10							
C	1	3	5	4	6	7	9	10	8	14	11	12		
v	1	2	3	4	5	6	7	8	9	10	11	12	13	14
D[v]	1	0	1	2	1	2	3	4	3	3	4	4	-	4
P[v]	2	-1	2	3	2	3	4	7	4	4	10	10	-	9

When backtracking, the *P* array is used to determine which candidates to be deleted from *C*. For instance, when backtracking from *U*^∗^ to *U*, we first delete last added candidates whose parent is 10 from *C* (11 and 12) and reset the values of these indices in the *D* and *P* arrays, then we delete the 10 vertex from *U*.

For further improvement, only valid candidates are kept in *C* to avoid redundant checking for valid candidates. We maintain three extra arrays to hold invalid candidates, the last added vertex to *U* when the candidate vertex became invalid, and the original index of the candidate vertex at *C*. We use information in these arrays to move these candidates back to their original indices in *C* when backtracking. Through extensive experiments, we noticed that the performance gain in the running time achieved from removing invalid candidates outweighs the extra cost associated with maintaining these arrays.

### Algorithm

Algorithm 1 shows pseudo-code for our algorithm. The recursive function takes a connected vertex set *U* and the set of candidate vertices. For each vertex *v* in the candidate set, it checks if it a valid extension and recursively calls the *EnumerateCIS* function. The algorithm invokes the *EnumerateCIS* function for each vertex in the graph.


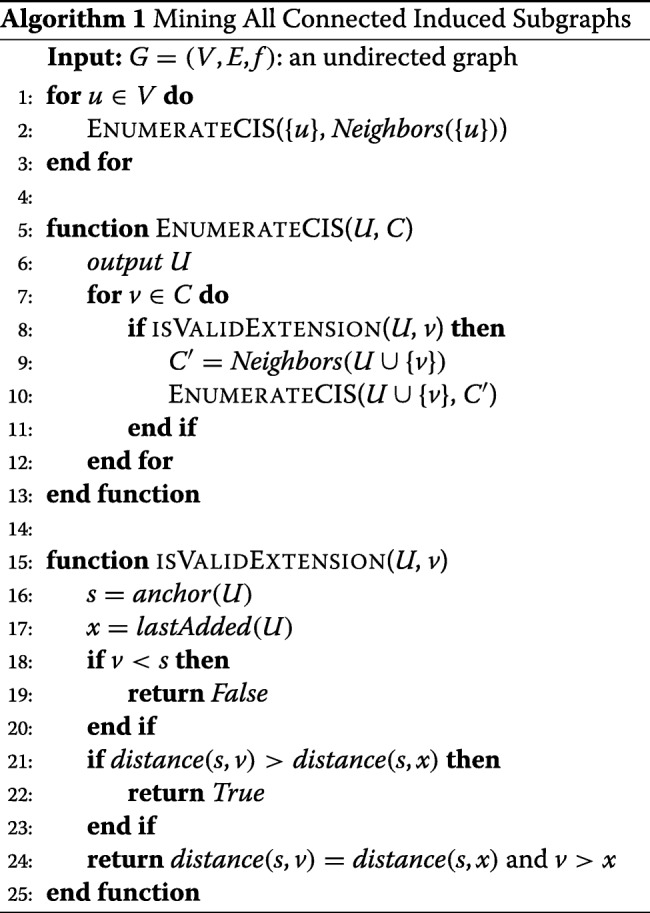


### Complexity analysis

An algorithm is said to be a linear-delay algorithm if it takes linear time, in terms of input size, to compute the next solution given a solution, or to detect that there are no more solutions. In our case, we consider the time the algorithm takes to generate the first child subgraph, given the parent subgraph. Clearly, our algorithm checks if a vertex is a valid neighbor of a subgraph in a constant time *O*(1) (Algorithm 1 line 8). It checks this condition for all vertices in the candidate set of a given connected vertex set. So, if there are no more solutions, the total delay is *O*(*N*) where *N*=|*V*|. In case there is a valid neighbor, the algorithm takes *O*(*Δ*) time to update the arrays of the data structure.

Note that the algorithm is a Depth First Search (DFS) algorithm which ensures that the space used is bounded by the depth of the search tree. This depth is bounded by the number of vertices in the graph since at each level we add one vertex. So the depth is linear in the number of nodes *N*, and we use 7 arrays of size *N* to keep track of which vertices are in the search node, their neighbors, and their distances to the *anchor* vertex. So, the algorithm uses a total extra space of *O*(*N*).

## Maximal cohesive subgraphs

In many applications, we are only interested in connected subgraphs that meet a user-defined constraint. Let $f:2^{V} \rightarrow \mathbb {R}$ denote a scoring function that quantifies vertex sets. Moreover, given a threshold *δ*, the anti-monotone constraint guarantees that if the score of a vertex set is at least *δ*, then score of each subset of the vertex set is also at least *δ*, i.e., *f*(*U*)≥*δ* ⇒ ∀*U*^∗^⊂*U*:*f*(*U*^∗^)≥*δ*

In this section, we assume that the vertices in the graph are annotated with features. This leads to the undirected attributed graph *G*=(*V*,*E*,*f*) where *V* is the set of vertices, *E* is the set of edges, and *f*:*V*→{0,1}^*d*^ is a function that maps vertices to *d*-dimensional binary vectors. We are interested in mining subsets of connected vertices that have similar features. A dimension *j* is a cohesive dimension for a vertex set(subgraph) if the value of the dimension is ‘1’ in all the binary vectors of the vertices of the set; *j* is cohesive for *U* if ∀*v*∈*U* | *f*(*v*)[*j*]=1. Let *A*(*U*) denote the set of cohesive dimensions for *U*.

Given a user-defined threshold *S*_*min*_, a subgraph *G*(*U*) is called *cohesive*, if the number of dimensions in *A*(*U*) is at least *S*_*min*_. The cohesive condition is an anti-monotone constraint where all the subgraphs of a cohesive graph are also cohesive. The set of all cohesive subgraphs for an attributed graph will have a large number of overlapping subgraphs since the subgraphs of a cohesive subgraph are also cohesive. To reduce redundancy in the output subgraphs, we require the subgraphs to be maximally cohesive. A subgraph is *maximal cohesive subgraph* if it does not have a supergraph that is cohesive, i.e., *G*(*U*) is *maximal cohesive* if $\nexists U^{*} \supset U, \ such \ that \ A(U^{*}) \geq S_{min}$.

**Problem Definition:** Given an attributed graph *G*=(*V*,*E*,*f*), and threshold *S*_*min*_, the problem of mining the set of **maximal cohesive subgraphs** is to enumerate the set:

$${\mathcal{M}}=\{M_{1},M_{2},M_{3},\cdots,M_{|{\mathcal{M}}|}\} $$

such that every $M_{i} \in \mathcal {M}$ is a maximal cohesive subgraph.

This problem can be addressed by employing the reverse search enumeration approach in algorithm 1 to enumerate all cohesive subgraphs and report only leaf search nodes that do not have any valid or invalid cohesive child nodes. For a highly-connected graph and a relaxed cohesive constraint, enumerating the entire search tree of all cohesive subgraphs is computationally expensive. In the following subsections, we describe pruning strategies to reduce the size of the enumeration tree by pruning entire search branches without missing any search nodes. The pruning strategies result in significant performance improvement.

### Nodes with a preceding covering sibling

Let *x* and *y* be two neighbors of *G*(*U*) such that *x* is closer to *anchor*(*U*) than *y* (*x*≺_*U*_*y*), and *G*(*U*∪{*x*}) and *G*(*U*∪{*y*}) are cohesive subgraphs with *A*(*U*∪{*y*})⊆*A*(*U*∪{*x*}), then none of them is a maximal cohesive subgraph, and any maximal subgraph that contains *G*(*U*∪{*x*}) will also contain *G*(*U*∪{*y*}), and vise versa. Moreover, *G*(*U*∪{*x*,*y*}) is also a cohesive subgraphs that can be reached from both *G*(*U*∪{*x*}) and *G*(*U*∪{*y*}), but is a valid child of only one of them. Note that since *A*(*U*∪{*y*})⊆*A*(*U*∪{*x*}), we get *A*(*U*∪{*x*,*y*})=*A*(*U*∪{*y*}).

In this case, we can prune the search branch rooted at one of the two subgraphs.

#### **Lemma 2**

Let *G*(*U*∪{*x*}) and *G*(*U*∪{*y*}) be two cohesive subgraphs, *x* is closer to *anchor*(*U*) than *y* (*x*≺_*U*_*y*), and *A*(*U*∪{*y*})⊆*A*(*U*∪{*x*}), then the search branch rooted at *G*(*U*∪{*y*}) can be safely pruned.

#### *Proof*

For a set of vertices *Z*⊆*V*∖{*x*∪*y*}, assume that *G*(*U*∪{*y*}∪*Z*) is a maximal cohesive subgraph. *G*(*U*∪{*y*}∪*Z*∪*x*) is a cohesive subgraph since *x* is connected to *U* and can be added to *G*(*U*∪{*y*}∪*Z*) without violating the attribute similarity constraint. This contradicts our assumption that *G*(*U*∪{*y*}∪*Z*) is a maximal cohesive subgraph. This proves that *G*(*U*∪{*y*}∪*Z*) is not a maximal cohesive subgraph. Moreover, *G*(*U*∪{*y*}∪*Z*∪*x*) is not a descendant of *G*(*U*∪{*y*}) since *x* is not valid extension once *y* is added to vertex set *U* because *x* is closer to *anchor*(*U*) than *y*. But all descendants of *G*(*U*∪{*y*}) can be expressed as *G*(*U*∪{*y*}∪*Z*). So none of the descendants of *G*(*U*∪{*y*}) will be a maximal cohesive subgraph. Therefore, it is safe to prune the search branch rooted at *G*(*U*∪{*y*}) without losing any maximal cohesive subgraphs. □

**Fig. 4:**
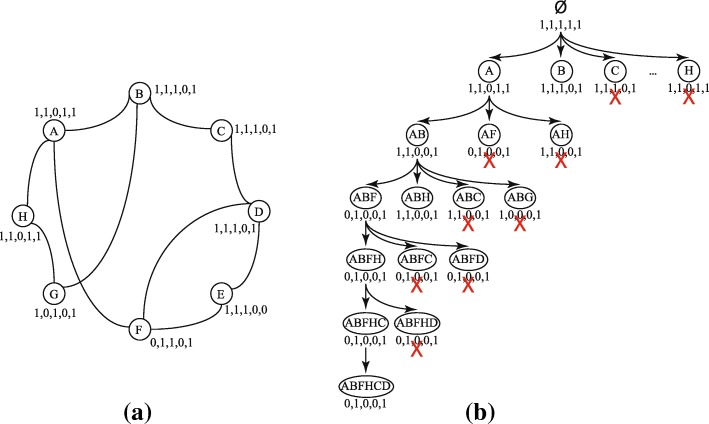
Enumerating cohesive subgraphs on a sample graph. **a** An example node attributed graph. **b** A portion of the traverse tree for attributed graph in Fig. 4 with *S*_*min*_=2. Crossed search nodes indicate pruned children

Figure 4a shows a sample attributed subgraph, and Fig. 4b shows a portion of the enumeration tree of this graph with *S*_*min*_=2. Search node {*A*,*F*} is pruned because it has a preceding sibling {*A*,*B*} where *A*({*A*,*B*,*F*}) = *A*({*A*,*F*}). Similarly, search nodes {*A*,*B*,*C*} and {*A*,*B*,*G*} are also pruned because they have a preceding sibling {*A*,*B*,*H*} where *A*({*A*,*B*,*H*,*C*}) = *A*({*A*,*B*,*C*}) and *A*({*A*,*B*,*H*,*G*}) = *A*({*A*,*B*,*G*}).

**Level One Pruning:** Pruning for level one (single vertex) is a special case, where *U*=*∅* and *A*(*U*)={1}^*d*^. If a vertex *x* in level one has a preceding connected vertex *y* with *A*(*y*)⊆*A*(*x*), then *y* can be safely pruned. In Fig. 4, the search branch rooted at *C* can be safely pruned because it is connected to *B* and and *A*(*C*)⊆*A*(*B*). Similarly, the branch rooted at *H* is pruned since *H* is connected to *A* and *A*(*H*)⊆*A*(*A*).

### Nodes with the same features as its parent

This pruning strategy handles a special case where the attributes of a child node are identical to those of the parent node. After sorting neighbors of *U*, if there is a child *U*^∗^ such that *A*(*U*) = *A*(*U*^∗^), then all succeeding neighbors can be pruned safely using the previous lemma, because their descendants will be enumerated under the *U*^∗^ search branch. Although it looks like that this pruning operation is theoretically redundant of the first operation, it saves practically the time needed to check if the siblings are covered by any proceeding one. So once we observe that there is a node with the same feathers as the parent node, there is no need to check whether the succeeding neighbors are covered by this node. We will show in the experiments section that this pruning technique improves the performance.

In Fig. 4b, search node {*A*,*B*,*F*,*H*} has same features as its parent, hence, all its succeeding siblings can be pruned.

### Algorithm

Algorithm 2 shows the pseudo code for our algorithm. The recursive function builds an enumeration tree. The result of this algorithm is the set of all maximal cohesive subgraphs ${\mathcal {M}}$. The main procedure is called for each cohesive vertex in the graph (lines 2–7). Sorting the neighbors according to the total order (closeness to *U*) is done in line 10. Checking for pruning the search node rooted at *U*∪{*v*_*i*_} is done in 15–19. Pruning the succeeding neighboring search nodes is done in lines 23–25. If there are no cohesive supergraphs of the current subgraph then it is added to the set of maximal subgraphs (lines 28–30).


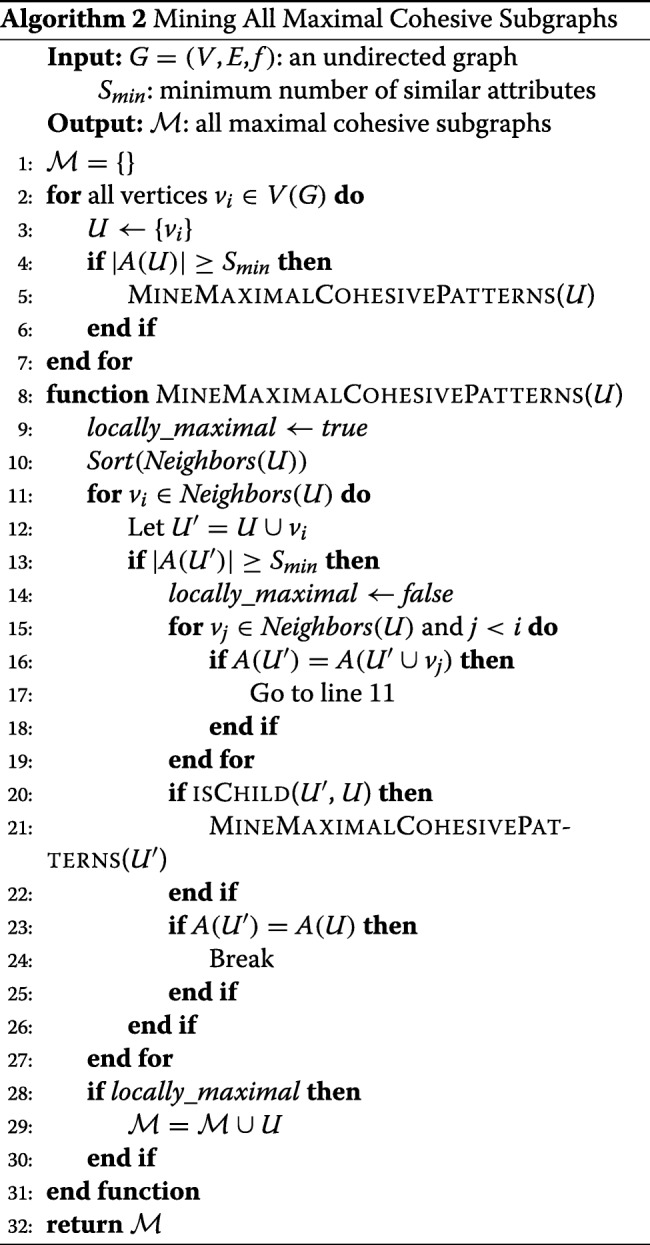


## Results

We compare the performance of the proposed approach for enumerating all connected induced subgraphs to that of three existing algorithms on random graphs with varying graph size and density. Moreover, we test the running time on real enzymes. Moreover, to test the performance of the proposed approach for mining maximal cohesive subgraphs, we evaluate the performance on a real protein-protein interaction network with gene dysregulation profile in 13 cancer types as attributes. All experiments were performed on a machine with Intel Xeon 2.40 GHz processor with 16 Gbytes main memory, running the Linux operating system. The two reverse search enumeration approaches were implemented in C++. The TGE algorithm is implemented in C and the BDDE algorithm in Perl as provided by their respective authors.

### Performance on random graphs

**Fig. 5:**
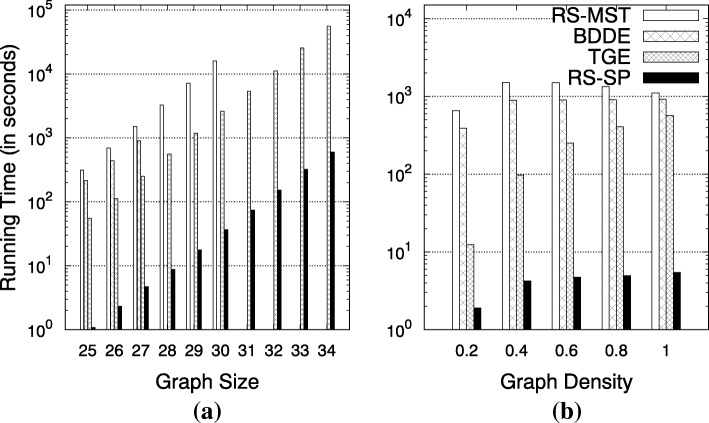
Running time on random graphs. **a** Varying graph size; graph density set to 0.6. **b** Varying graph density; graph size set to 27

We generated random graphs with varying numbers of nodes and density. Figure 5a shows the running times on graphs with varying size while keeping the density at 0.6. Figure 5b shows the running times on random graphs with varying density while the number of vertices was set to 27. We can see that RS-SP runs about one order of magnitude faster. We can see that our proposed algorithm is at least an order of magnitude faster than the best competing algorithm (TGE) and two orders of magnitude faster that the BDDE and RS-MST algorithms. For graphs with larger number of nodes (>28), the BDDE algorithm uses too much memory and crashes after 1 h. For larger graphs (>31), the RS-MST did not finish the enumeration task in 27 h.

### Performance on real data

We tested our algorithm on real chemical graphs downloaded from the network repository [16]. We compared against the TGE algorithm since it is the fastest among the competing algorithms. We ran both algorithms on ten graphs for which the running time is less than nine hours. For larger graphs, it takes days before we could get any results. Table 3 shows the running time of the TGE and RS-SP algorithms; RS-SP is several times faster than the TGE algorithm. Due to the nature of chemical compounds, most atoms (nodes) have a degree of at most 8 (maximal valence of atoms), and thus large chemical graphs are not dense. For these sparse graphs, the speedup is not high.

**Table 3:** Running time on real enzyme graphs

Graph ID	|*V*|	*ρ*	|*CIS*(*G*)|	*TGE*	RS-SP
			(in millions)		
502	36	0.116	53.4	10	1
522	37	0.123	2,376.7	438	54
31	38	0.115	4,470.0	850	119
108	38	0.117	3,125.8	566	69
23	39	0.109	713.7	111	15
274	40	0.094	1,723.2	291	45
303	41	0.101	22,534.5	4935	696
513	41	0.112	31,041.1	5017	715
530	42	0.096	44,684.8	7510	1117
500	43	0.109	184,636.9	31,130	4618

### Cohesive subnetworks

We use the BIOGRID protein-protein interaction network (PPI) (version 3.4.160; May 2018) that has 287,970 interactions among 21,429 genes [17]. For attribute data, we used the gene dysregulation profile in 13 cancers. The dataset was generated from the gene and miRNA expression data of 13 tumor types and matched normal samples [18]. On average each cancer dataset had 2380 dysregulated genes and each gene was dysregulated in 3.4 cancers. We ran the algorithm with all the pruning techniques on the attributed BIOGRID network for varying minimum support. The algorithm was extremely fast finishing in less than one second for *S*_*min*_≥6, and for *S*_*min*_=2, and 1 it took 21 and 74 seconds, respectively.

### Effectiveness of pruning techniques

**Fig. 6:**
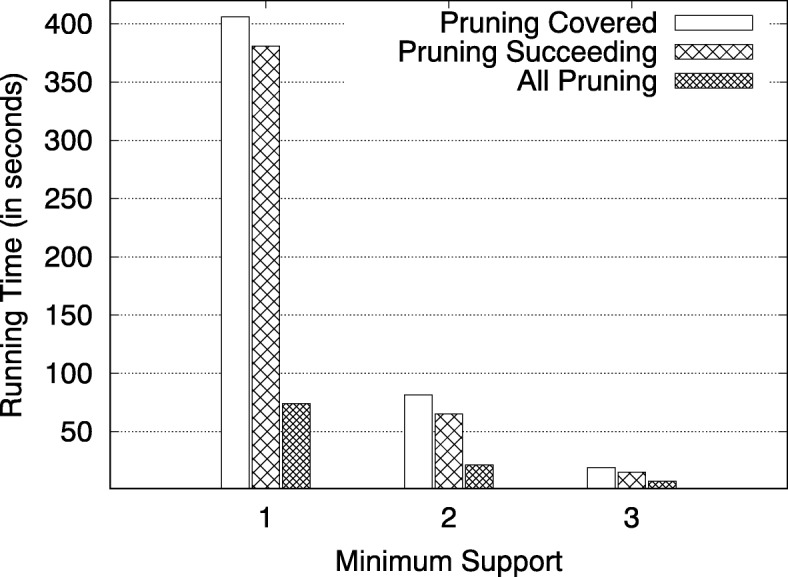
Effectiveness of Pruning Techniques

To show the impact of the pruning techniques on the running time, we turned off the pruning techniques in the algorithm one at a time. Figure 6 shows the impact of the pruning techniques. For 1≤*S*_*min*_≥3, the algorithm without any pruning did not finish in 50 h, resulting in more than 400 speed up for each of the pruning techniques.

### Maximal cohesive subgraphs analysis

Table 4 shows the topological properties and biological enrichment analysis for the genesets in the reported maximal cohesive patterns. As we decrease *S*_*min*_ (relaxing the constraint), the average size of reported subgraphs, $\overline {N}$, increases. Moreover, the number of subgraphs increases but then decreases when *S*_*min*_=4 the subgraphs increase in size.

**Table 4:** Enrichment analysis of maximal cohesive subgraphs

*S*_*min*_	*N*	$\overline {N}$	$\overline {Density}$	*H* *a* *l* *l* *m* *a* *r* *k* *%*	*K* *E* *G* *G* *%*	*O* *n* *c* *o* *%*	*D* *i* *s* *G* *e* *N* *e* *t* *%*
1	28	798.6	0.185	68	71	79	93
2	260	124.5	0.19	65	77	59	91
3	642	58.5	0.147	67	72	65	89
4	816	43.5	0.123	76	78	74	92
5	705	37.0	0.106	83	82	80	96
6	429	31.2	0.104	88	86	84	99
7	183	25.9	0.113	89	88	84	99
8	72	20.7	0.125	92	86	89	99
9	32	15.3	0.154	91	78	81	100

We performed biological enrichment analysis of the reported patterns. We used the Molecular Signatures Database (MSigDB) [19, 20] and the DisGeNET human gene-disease associations database [21] for assessing the enrichment of the genes in these reported patterns with these signatures. If a biological annotation is overrepresented in the reported subgraph’s genes, the subgraph pattern is considered as enriched. The overrepresentation test is modeled as a hybergeometric test (with *pvalue*=0.05) and we checked for enrichment in the following collections:

1. Hallmark signatures: gene sets that represent biological processes and display coherent expression.
2. KEGG signatures: gene sets derived from the KEGG pathway database.
3. Oncogenic signatures: gene sets of dysregulated cellular pathways in caner.
4. DisGeNET curated human gene-disease associations database.

Table 4 shows the percentage of patterns that are biologically enriched with different biological signatures. Some patterns are enriched with several signatures and some signatures are enriched in the genes of more than one pattern.

Table 5 shows the biological signatures that were enriched the most in the reported patterns for *S*_*min*_=9 along with the number of patterns each signature was enriched in.

**Table 5:** Top enriched signatures in the cohesive subgraphs; *S*_*min*_=9

Hallmark gene sets	N	KEGG Pathways	N
G2M_CHECKPOINT	22	CELL_CYCLE	20
E2F_TARGETS	22	OOCYTE_MEIOSIS	20
MITOTIC_SPINDLE	20	PROGESTERONE_	17
		OOCYTE_MATURATION	
MYC_TARGETS_V1	7	UBIQUITIN_MEDIATED_	8
		PROTEOLYSIS	
TNFA_SIGNALING_VIA_NFKB	4	P53_SIGNALING_	5
		PATHWAY	
MTORC1_SIGNALING	2	HYPERTROPHIC_	2
		CARDIOMYOPATHY_HCM	
Oncogenic Signatures	N	Gene-Disease Association	N
CSR_LATE_UP.V1_UP	20	Mammary Neoplasms	20
GCNP_SHH_UP_LATE.V1_UP	20	leukemia	18
RB_P107_DN.V1_UP	20	Salivary Gland Neoplasms	13
CORDENONSI_YAP_CONSERVED	20	Polycystic Ovary Syndrome	5
RPS14_DN.V1_DN	18	Cerebellar Hypoplasia	4
GCNP_SHH_UP_EARLY.V1_UP	17	Adenoid Cystic Carcinoma	3

## Discussion and conclusions

We have proposed a new reverse search algorithm for enumerating all connected induced subgraphs in a single graph. Furthermore, we employed the proposed techniques for mining maximal connected subgraphs that satisfy a constraint defined over the attributes of the vertices. Leveraging on the order in which the subgraphs are enumerated, we proposed two pruning strategies that drastically reduce the running time of the algorithm by pruning search branches that will not result in maximal subgraphs. Experiments on both synthetic and real datasets demonstrate the effectiveness of the proposed approaches. Enrichment analysis of the reported protein-protein subnetworks whose genes are dysregulated in a number of cancers reveals that these subnetworks are biologically significant. Future work includes developing a parallel implementation of the algorithm and designing pruning strategies for real-valued vertex attributes.

## Abbreviations

BIOGRID: The biological general repository for interaction datasets
DisGeNET: Human gene-disease associations database
MSigDB: Molecular signatures database
PPI: Protein-protein interaction
